# Proteomic analysis of *Citrus sinensis* roots and leaves in response to long-term magnesium-deficiency

**DOI:** 10.1186/s12864-015-1462-z

**Published:** 2015-03-31

**Authors:** Hao-Yang Peng, Yi-Ping Qi, Jinwook Lee, Lin-Tong Yang, Peng Guo, Huan-Xin Jiang, Li-Song Chen

**Affiliations:** College of Resource and Environmental Science, Fujian Agriculture and Forestry University, Fuzhou, 350002 China; College of Life Science, Fujian Agriculture and Forestry University, Fuzhou, 350002 China; Institute of Horticultural Plant Physiology, Biochemistry, and Molecular Biology, Fujian Agriculture and Forestry University, Fuzhou, 350002 China; Institute of Materia Medica, Fujian Academy of Medical Sciences, Fuzhou, 350001 China; Department of Horticultural Science, Kyungpook National University, Daegu, 702-701 ROK; Fujian Key Laboratory for Plant Molecular and Cell Biology, Fujian Agriculture and Forestry University, Fuzhou, 350002 China; The Higher Educational Key Laboratory of Fujian Province for Soil Ecosystem Health and Regulation, Fujian Agriculture and Forestry University, Fuzhou, 350002 China

**Keywords:** *Citrus sinensis*, Magnesium (Mg)-deficiency, Photosynthesis, Proteomics, Respiration, Reactive oxygen species

## Abstract

**Background:**

Magnesium (Mg)-deficiency is frequently observed in *Citrus* plantations and is responsible for the loss of productivity and poor fruit quality. Knowledge on the effects of Mg-deficiency on upstream targets is scarce. Seedlings of ‘Xuegan’ [*Citrus sinensis* (L.) Osbeck] were irrigated with Mg-deficient (0 mM MgSO_4_) or Mg-sufficient (1 mM MgSO_4_) nutrient solution for 16 weeks. Thereafter, we first investigated the proteomic responses of *C. sinensis* roots and leaves to Mg-deficiency using two-dimensional electrophoresis (2-DE) in order to (*a*) enrich our understanding of the molecular mechanisms of plants to deal with Mg-deficiency and (*b*) understand the molecular mechanisms by which Mg-deficiency lead to a decrease in photosynthesis.

**Results:**

Fifty-nine upregulated and 31 downregulated protein spots were isolated in Mg-deficient leaves, while only 19 upregulated and 12 downregulated protein spots in Mg-deficient roots. Many Mg-deficiency-responsive proteins were involved in carbohydrate and energy metabolism, followed by protein metabolism, stress responses, nucleic acid metabolism, cell wall and cytoskeleton metabolism, lipid metabolism and cell transport. The larger changes in leaf proteome *versus* root one in response to Mg-deficiency was further supported by our observation that total soluble protein concentration was decreased by Mg-deficiency in leaves, but unaffected in roots. Mg-deficiency had decreased levels of proteins [i.e. ribulose-1,5-bisphosphate carboxylase (Rubisco), rubisco activase, oxygen evolving enhancer protein 1, photosynthetic electron transfer-like protein, ferredoxin-NADP reductase (FNR), aldolase] involved in photosynthesis, thus decreasing leaf photosynthesis. To cope with Mg-deficiency, *C. sinensis* leaves and roots might respond adaptively to Mg-deficiency through: improving leaf respiration and lowering root respiration, but increasing (decreasing) the levels of proteins related to ATP synthase in roots (leaves); enhancing the levels of proteins involved in reactive oxygen species (ROS) scavenging and other stress-responsive proteins; accelerating proteolytic cleavage of proteins by proteases, protein transport and amino acid metabolism; and upregulating the levels of proteins involved in cell wall and cytoskeleton metabolism.

**Conclusions:**

Our results demonstrated that proteomics were more affected by long-term Mg-deficiency in leaves than in roots, and that the adaptive responses differed between roots and leaves when exposed to long-term Mg-deficiency. Mg-deficiency decreased the levels of many proteins involved in photosynthesis, thus decreasing leaf photosynthesis.

**Electronic supplementary material:**

The online version of this article (doi:10.1186/s12864-015-1462-z) contains supplementary material, which is available to authorized users.

## Background

Magnesium (Mg), a common constituent in many minerals, comprising 2% of the Earth’s crust, is an essential macronutrient used in large amount by plants for their normal growth and development. Mg-deficiency is a widespread nutritional disorder, affecting productivity and quality in agriculture [1]. Mg is taken up by plants in the form of divalent Mg^2+^ (the form of dissolved Mg in the soil solution). The binding strength of Mg^2+^ to the soil colloids is low, because Mg^2+^ has a large hydrated radius. Therefore, Mg is highly prone to leaching, particularly in acidic soils with low cation exchange capacity. Leaching is considered as a key factor affecting Mg availability for roots [2]. Mg-deficiency can also be induced by high levels of competing elements, such as potassium (K) and calcium (Ca), which strongly inhibit Mg uptake by plants [1,3].

Since Mg is mobile within the plant, Mg-deficiency symptoms first appear on lower and older leaves. The typical symptom of Mg-deficiency is leaf interveinal chlorosis [4]. Although Mg-deficiency symptoms are well described in plant shoots, the responses of both plant root growth and biomass allocation between roots and shoots to Mg-deficiency is more variable. Previous study showed a less severe impact on root growth or shoot growth, depending on the plant species and the system used to create Mg-deficiency [1]. Mg is the central component of the chlorophyll (Chl) molecule and plays a crucial role in photosynthesis such as Chl biosynthesis, photochemical reactions, CO_2_ fixation and stomata functioning [1,5]. Mg-deficiency-induced inhibition of photosynthesis is a wide phenomenon observed in many plant species [4-6]. In addition, Mg also act as a cofactor and allosteric modulator for more than 300 enzymes including ribulose-1,5-bisphosphate carboxylase (Rubisco), ATPase, protein kinases, RNA polymerase, phosphatases and glutathione synthase [1,4]. Therefore, Mg also functions in many other physiological processes, such as respiration [7], glycolysis, tricarboxylic acid (TCA) cycle [8], energy transfer *via* adenosine triphosphate [9], carbohydrate partitioning between source and sink organs [1,10], reactive oxygen species (ROS) formation and related photooxidative damage [5,11], protein biosynthesis, and the formation of DNA and RNA [3]. Accordingly, a number of studies have investigated the effects of Mg-deficiency on Chl synthesis, transport and utilization of photosynthates [1], photochemical reactions, CO_2_ fixation [2,4-6], respiration, TCA cycle [7,8], and ROS metabolism [4,5,11].

Physiological and molecular biological analyses have allowed us to draw a picture of abiotic stress responses in various plants. Although the physiological targets upon Mg-deficiency have been reported by many workers in various plants [1,4], the knowledge of upstream molecular targets is very limited until recently. Hermans et al. [12,13] investigated the transcriptomic responses to 4, 8 and 24 h Mg-deficiency, or long-term (1 week) Mg-deficiency and restoration in *Arabidopsis thaliana* roots and leaves and identified numerous target genes involved in the circadian clock, the redox control of the cell and the protection of the photosynthetic apparatus. It is worth mentioning that the responses of global transcriptomics to Mg-deficiency were asynchronized, with a higher number of differentially expressed genes after 4 or 8 h in roots and after 28 h or 1 week in leaves. While these data are very useful, the abundances of mRNAs does not necessarily correspond directly the abundances of their corresponding proteins. The level of a protein depends not only on transcription rates of the gene, but also on nuclear export and mRNA localization, transcript stability, translational regulation and protein degradation. Indeed, there is considerable variability on protein level *versus* mRNA level [14]. Since biological processes are ultimately controlled by proteins, a proteomic analysis is necessary to get a better understanding of the plant responses to Mg-deficiency. To our knowledge, little information is available on the changes of protein profile under Mg-deficiency in plant roots and leaves [1].

In China, Mg-deficiency is frequently observed in *Citrus* plantations and is responsible for the loss of productivity and poor fruit quality [5]. Although the effects of Mg-deficiency on *Citrus* CO_2_ assimilation, photosynthetic electron transport and antioxidant system, carbohydrates and organic acid metabolism have been investigated by a few researchers [5,6,8], the knowledge of upstream targets is scarce. In the present study, we first investigated the proteomic responses of *Citrus* leaves and roots to Mg-deficiency using two-dimensional electrophoresis (2-DE) in order to (*a*) enrich our understanding of the molecular mechanisms of plants to deal with Mg-deficiency and (*b*) understand the molecular mechanisms by which Mg-deficiency lead to a decrease in CO_2_ assimilation.

## Results

### Seedling growth, leaf, stem and root Mg concentration

**Figure 1:**
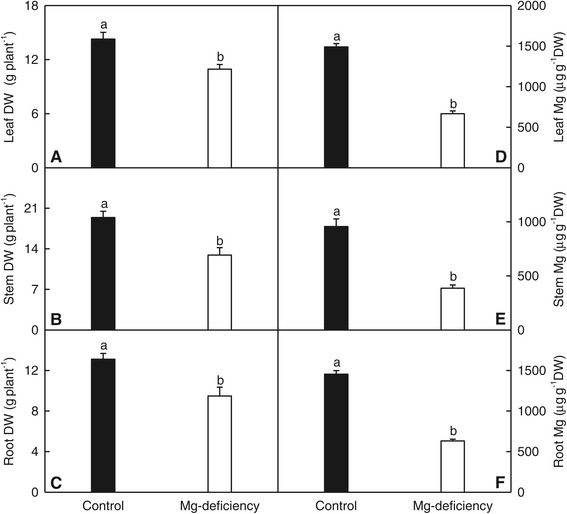
**Effects of Mg-deficiency on growth and Mg concentration in leaves, stems and roots. (A-C)** Leaf, stem and root DW. **(D-F)** Leaf, stem and root Mg concentration. Bars represent means ± SE (*n* = 10 except for 9 for leaf, stem and root DW of Mg-deficient seedlings). Different letters above the bars indicate a significant difference at *P* < 0.05.

Plant treated with 0 mM Mg displayed decreased leaf, stem and root dry weight (DW) and lower concentration of Mg in leaves, stems and roots (Figure 1), and leaf Mg concentration was much lower than the normal range [15]. Based on these results, plants that did not receive Mg are considered Mg-deficient, and those treated with 1 mM Mg are considered Mg-sufficient (control).

### Leaf gas exchange, leaf and root respiration and total soluble protein concentration

**Figure 2:**
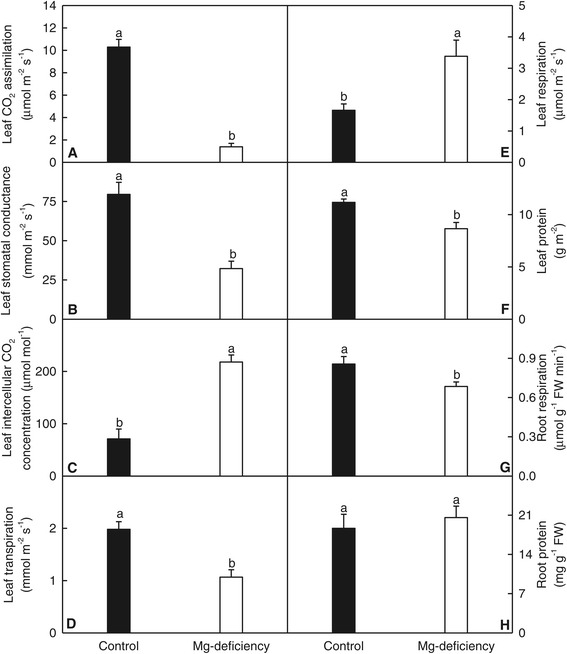
**Effects of Mg-deficiency on leaf gas exchange, root respiration, and root and leaf total soluble protein. (A-E)** Leaf CO_2_ assimilation, stomatal conductance, intercellular CO_2_ concentration, transpiration and respiration. **(F)** Leaf total soluble protein. **(G)** Root respiration. **(H)** Root total soluble protein. Bars represent means ± SE (*n* = 5 except for 8 for leaf CO_2_ assimilation, stomatal conductance, intercellular CO_2_ concentration and transpiration of control and Mg-deficient seedlings, respectively). Different letters above the bars indicate a significant difference at *P* < 0.05.

Compared with controls, Mg-deficient leaves had lower CO_2_ assimilation (Figure 2A), stomatal conductance (Figure 2B) and transpiration (Figure 2D), and higher intercellular CO_2_ concentration (Figure 2C).

Mg-deficient leaves displayed increased dark respiration (Figure 2E) and decreased concentration of total soluble proteins (Figure 2F), while Mg-deficient roots had decreased respiration (Figure 2G) and unchanged concentration of total soluble proteins (Figure 2H).

### Profiles of differentially expressed proteins

**Figure 3:**
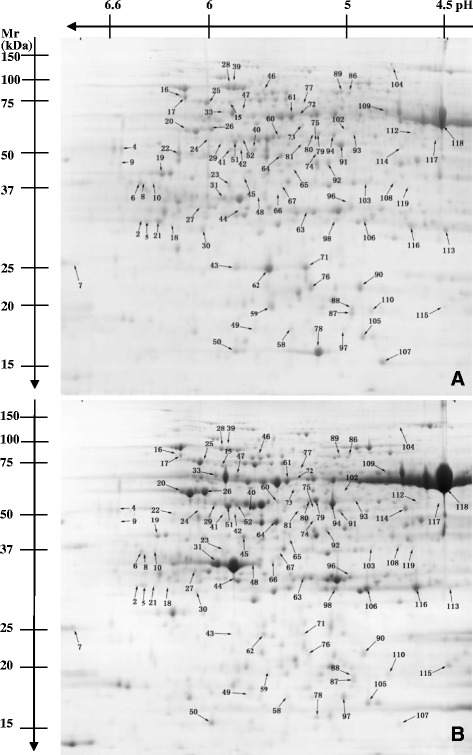
**Representative gel images of proteins in Mg-deficient (A) and control (B) leaves.** Proteins were separated in the first dimension on an IPG strip pH 3–7 and in the second dimension on a 12% slab gel, followed by colloidal Coomassie Brilliant G-250. An equal amount (1.5 mg) of total protein extracts was loaded in each gel.

**Figure 4:**
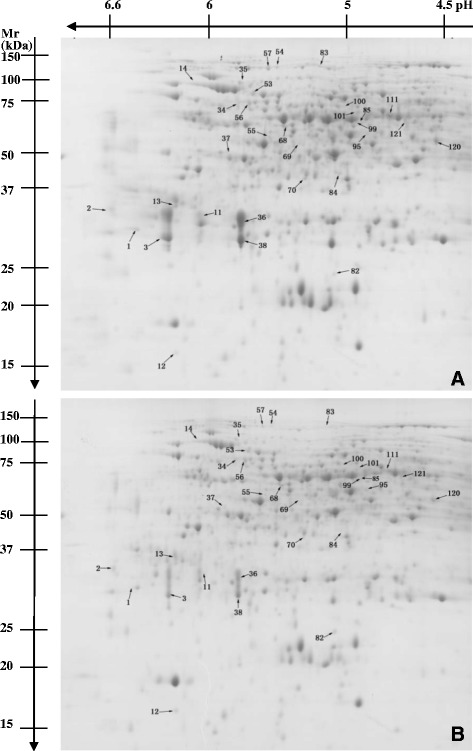
**Representative gel images of proteins in Mg-deficient (A) and control (B) roots.** Proteins were separated in the first dimension on an IPG strip pH 3–7 and in the second dimension on a 12% slab gel, followed by colloidal Coomassie Brilliant G-250. An equal amount (1.5 mg) of total protein extracts was loaded in each gel.

2-DE was performed to compare the protein profiles between control and Mg-deficient roots and leaves. In order to get credible results, the experiments were performed in 3 biological replicates. After Coomassie Brilliant Blue G-250 staining, more than 900 clear and reproducible spots were detected on each gel (Figures 3 and 4). Comparative analysis of the 2-DE maps of control and Mg-deficient leaves was performed by PDQuest 8.0.1 software. A protein spot was considered differentially expressed when the protein had both a fold change of more than 2 and a *P*-value less than 0.05. Based on the two criteria, 90 (ca.10.0% of the total protein spots) differentially expressed protein spots were detected in Mg-deficient leaves with high confidence, 59 of which displayed increased and 31 displayed decreased level under Mg-deficient condition; and 32 (ca. 3.6% of the total protein spots) differentially expressed protein spots were detected in Mg-deficient roots with high confidence, including 20 protein spots that were upregulated and 12 protein spots that were downregulated by Mg-deficiency.

**Figure 5:**
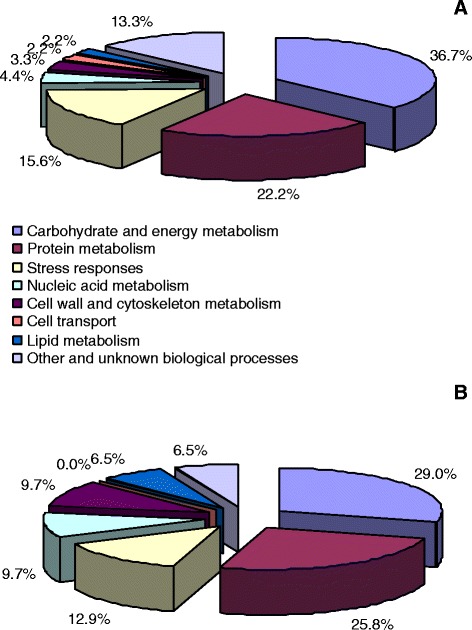
**Functional classification of the differentially expressed protein spots in leaves (A) and roots (B).**

All these differentially expressed protein spots were excised from the 2-DE gels and submitted to MALDI-TOF/TOF-MS and LTQ-ESI-MS/MS. In total, 90 and 31 protein spots were identified in Mg-deficient leaves and roots, respectively. The database searching results are presented in Tables 1 and 2. According to the biological functional properties, these differentially expressed protein spots in Mg-deficient leaves were classified into the following functional categories: carbohydrate and energy metabolism (36.7%), protein metabolism (22.2%), stress responses (15.6%), nucleic acid metabolism (4.4%), cell wall and cytoskeleton metabolism (3.3%), cell transport (2.2%), lipid metabolism (2.2%), other and unknown biological processes (13.3%) (Table 1 and Figure 5); and theses protein spots in Mg-deficient roots were involved in carbohydrate and energy metabolism (29.0%), protein metabolism (25.8%), stress responses (12.9%), nucleic acid metabolism (9.7%), cell wall and cytoskeleton metabolism (9.7%), lipid metabolism (6.5%), other and unknown biological processes (6.5%) (Table 2 and Figure 5).

**Table 1:** **List of differentially expressed proteins identified using MALDI-TOF/TOF-MS in magnesium (Mg)-deficient** ***Citrus sinensis*** **leaves**

**Spot no.** ^**a**^	**Accession no.** ^**b**^	**Protein identity**	**Organism**	**MW (kDa)**	**MP/SC**	**Score**	**Ratio** ^**c**^
***Carbohydrate and energy metabolism***							
L118	gi|4206520	Ribulose 1,5-bisphosphate carboxylase, partial	*Severinia buxifolia*	49.67	23/86	450	0.267
L20	gi|19992	Ribulose bisphosphate carboxylase activase	*Nicotiana tabacum*	25.91	14/92	418	0.478
L24	gi|290766483	Rubisco activase	*Glycine max*	52.39	19/90	315	0.051
L26	gi|19992	Ribulose bisphosphate carboxylase activase	*N. tabacum*	25.91	14/95	398	0.259
L31	gi|19992	Ribulose bisphosphate carboxylase activase	*N. tabacum*	25.91	15/94	435	0.170
L51	gi|100380	Ribulose-bisphosphate carboxylase activase		25.91	13/96	397	0.388
L52	gi|19992	Ribulose-bisphosphate carboxylase activase	*N. tabacum*	25.91	11/98	264	0.190
L48	gi|326467059	Oxygen evolving enhancer protein 1	*Litchi chinensis*	35.17	14/96	288	0.020
L115	gi|89475526	Photosynthetic electron transfer-like protein	*Panax ginseng*	19.64	5/103	93	0.412
L119	gi|158145455	Putative ferredoxin-NADP reductase	*Solanum peruvianum*	18.02	11/98	111	0.325
L114	gi|77540212	Glyceraldehyde-3-phosphate dehydrogenase B subunit	*Glycine max*	48.20	13/96	167	0.323
L80	gi|313585890	Phosphoglycerate kinase	*Nicotiana benthamiana*	50.05	14/93	357	0.239
L93	gi|313585890	Phosphoglycerate kinase	*N. benthamiana*	50.05	11/99	281	0.346
L94	gi|1161600	Phosphoglycerate kinase	*N. tabacum*	50.15	20/90	565	0.457
L106	gi|255567325	Carbonic anhydrase, putative	*Ricinus communis*	35.64	4/106	83	0.295
L44	gi|295687231	Triosephosphate isomerase	*Gossypium hirsutum*	33.10	7/102	121	0
L108	gi|330252068	Fructose-bisphosphate aldolase, class I	*Arabidopsis thaliana*	41.78	10/98	138	0.442
L67	gi|332196500	Putative lactoylglutathione lyase, chloroplast	*A. thaliana*	39.14	17/82	58	0
L81	gi|642352	Malate dehydrogenase (NADP)	*Spinacia oleracea*	47.46	1/98	23	2.320
L89	gi|255579273	Succinate dehydrogenase, putative	*R. communis*	68.46	23/87	293	2.438
L104	gi|285309967	Aconitate hydratase 3	*Citrus clementina*	98.04	30/78	330	3.176
L112	gi|255578100	Dihydrolipoamide succinyltransferase component of 2-oxoglutarate dehydrogenase, putative	*R. communis*	50.84	9/99	115	2.262
L72	gi|289600010	2-Phospho-D-glycerate hydrolase	*Citrus trifoliata*	47.76	5/98	449	3.389
L86	gi|332190370	2,3-Bisphosphoglycerate-independent phosphoglycerate mutase 1	*A. thaliana*	60.54	20/90	314	3.775
L60	gi|20336385	Alpha-amylase	*Citrus reticulata*	17.05	9/101	220	4.083
L77	gi|7671230	ADP-glucose pyrophosphorylase catalytic subunit	*Perilla frutescens*	57.44	16/83	63	2.080
L61	gi|56784991	Putative ATP synthase beta subunit	*Oryza sativa Japonica Group*	45.88	22/88	706	0.280
L65	gi|332191230	ATP synthase gamma chain 2	*A. thaliana*	42.65	7/102	88	0.101
L33	gi|113952607	ATP synthase CF1 alpha subunit, chloroplastic	*C. sinensis*	55.45	32/78	781	0.205
L47	gi|122166198	ATP synthase subunit alpha, chloroplastic		55.45	30/80	632	0.218
L40	gi|41350585	Putative adenosine kinase	*Populus tremula × Populus alba*	24.99	6/104	82	2.174
L105	gi|255571035	Nucleoside diphosphate kinase, putative	*R. communis*	16.30	3/104	116	3.070
L117	gi|33149683	Alcohol dehydrogenase	*Dianthus caryophyllus*	41.23	8/101	284	100
***Protein metabolism***							
L79	gi|255540493	Elongation factor tu, putative	*Ricinus communis*	50.09	20/90	505	0.357
L25	gi|806808	Chaperonin precursor (chloroplast)	*Pisum sativum*	62.95	13/97	177	100
L71	gi|3098188	Small ribosomal protein 4, partial (chloroplast)	*Plagiomnium affine*	22.29	12/97	57	24.429
L88	gi|193788982	Ribosomal protein S3	*Trifolium subterraneum*	24.57	9/100	51	2.067
L109	gi|12802327	Mitochondrial processing peptidase beta subunit	*Cucumis melo*	58.85	17/91	269	100
L41	gi|332005228	F-box domain-containing protein	*A. thaliana*	50.19	15/94	57	3.351
L8	gi|31433129	F-box family protein, putative, expressed	*O. sativa Japonica Group*	34.58	11/98	57	100
L9	gi|334302804	Putative F-box/kelch-repeat protein		42.48	16/94	67	100
L62	gi|12324823	Putative RING zinc finger protein	*A. thaliana*	12.29	11/99	61	21.438
L4	gi|89274062	Cysteine proteinase	*Platycodon grandiflorus*	50.77	6/101	91	3.780
L6	gi|89274062	Cysteine proteinase	*P. grandiflorus*	50.77	10/96	63	17.176
L45	gi|255538698	Proteasome subunit alpha type, putative	*R. communis*	30.35	11/98	249	3.490
L49	gi|332656653	Putative cathepsin B-like cysteine protease	*A. thaliana*	39.32	8/100	148	100
L58	gi|332656653	Putative cathepsin B-like cysteine protease	*A. thaliana*	39.32	9/98	156	7.633
L27	gi|9280680	F2E2.12	*A. thaliana*	12.88	9/99	55	0.476
L98	gi|193848487	Putative skp1 protein	*Brachypodium distachyon*	18.69	10/100	57	0.442
L75	gi|121489623	Putative glutamine synthetase	*P. sativum*	39.02	10/99	136	7.333
L91	gi|121489623	Putative glutamine synthetase	*P. sativum*	39.02	10/100	196	2.087
L102	gi|297843044	S-adenosylmethionine synthetase	*Arabidopsis lyrata subsp. lyrata*	41.43	18/91	230	100
L113	gi|18150415	Glutathione S-transferase	*Allium cepa*	23.43	1/96	55	2.873
***Stress responses***							
L50	gi|2274917	Cu/Zn superoxide dismutase	*Citrus sinensis*	12.78	8/102	259	100
L78	gi|2274917	Cu/Zn superoxide dismutase	*C. sinensis*	12.78	6/102	127	22.111
L63	gi|221327589	Ascorbate peroxidase 2	*Citrus maxima*	27.56	18/91	230	3.426
L96	gi|189476292	Ascorbate peroxidase	*C. maxima*	22.65	10/97	313	100
L97	gi|186920323	Chloroplast Cu/Zn superoxide dismutase	*Hevea brasiliensis*	6.92	6/80	219	0.410
L74	gi|223543700	Aldo/keto reductase, putative	*R. communis*	37.98	8/99	103	3.418
L103	gi|255543887	Aldo-keto reductase, putative	*R. communis*	34.84	5/104	102	7.957
L28	gi|20559	Heat shock protein 70	*Petunia x hybrida*	70.74	33/77	523	2.595
L39	gi|211906496	Heat shock protein 70	*Gossypium hirsutum*	71.17	34/76	456	3.219
L46	gi|300265	HSP68 = 68 kda heat-stress DnaK homolog	*Lycopersicon peruvianum*	62.34	15/94	79	3.960
L59	gi|259123935	CII small heat shock protein 1	*Prunus salicina*	17.52	6/10	102	314.482
L76	gi|116643152	Stress-related protein	*Citrus sinensis*	17.59	14/94	310	6.476
L90	gi|116643152	Stress-related protein	*Citrus sinensis*	17.59	14/95	382	4.825
L116	gi|332661276	Late embryogenesis abundant (LEA) protein	*A. thaliana*	37.94	12/98	47	0.441
***Nucleic acid metabolism***							
L5	gi|89258208	Maturase, partial (mitochondrion)	*Nepenthes sp. ‘Kosobe’*	67.36	16/94	54	3.741
L17	gi|115466830	Os06g0187000 protein	*Oryza sativa Japonica Group*	91.71	17/92	56	3.776
L22	gi|4063759	Mutator-like transposase	*A. thaliana*	80.331	18/92	65	14.059
L73	gi|255560725	Dead box ATP-dependent RNA helicase	*R. communis*	46.812	27/81	393	2.228
***Cell wall and cytoskeleton metabolism***							
L16	gi|56603655	Myosin class 11-1	*Adiantum capillus-veneris*	173.69	27/82	63	2.339
L21	gi|216296850	UGT1 (UDP-glucosyltransferase)	*Pueraria montana var. lobata*	52.19	13/97	56	23.600
L107	gi|38260664	Pollen coat oleosin-glycine rich protein	*Olimarabidopsis pumila*	47.00	12/98	39	100
***Cell transport***							
L87	gi|108707728	Mitochondrial import inner membrane translocase subunit Tim17/Tim22/Tim23 family protein, putative	*Oryza sativa Japonica Group*	18.36	1/96	34	4.384
L110	gi|297793335	ATYKT62	*A. lyrata subsp. lyrata*	22.64	7/52	51	100
***Lipid metabolism***							
L43	gi|108707070	Type I inositol-1,4,5-trisphosphate 5-phosphatase CVP2, putative	*Oryza sativa Japonica*	54.41	14/94	56	100
L19	gi|255545978	Cytochrome P450, putative	*R. communis*	77.12	15/95	48	2.320
***Other and unknown biological processes***							
L10	gi|163943829	*Ent*-kaurene synthase	*Luziola fluitans*	42.96	13/96	53	100
L66	gi|255547472	4-Nitrophenylphosphatase, putative	*R. communis*	39.69	17/92	167	0
L29	gi|825532	Orf	*Pseudotsuga menziesii*	17.00	8/99	51	0.350
L23	gi|2582665	Thi	*C. sinensis*	37.57	21/89	197	8.333
L15	gi|108711987	Streptomyces cyclase/dehydrase family protein, expressed	*Oryza sativa Japonica Group*	57.34	15/76	60	2.595
L30	gi|147855631	Hypothetical protein VITISV_019248	*Vitis viniferai*	35.05	12/98	346	2.365
L64	gi|302810346	Hypothetical protein SELMODRAFT_182694	*Selaginella moellendorffii*	47.01	9/101	215	0.480
L42	gi|147812626	Hypothetical protein VITISV_007608	*Vitis vinifera*	27.14	8/101	167	11.185
L18	gi|326530266	Predicted protein	*Hordeum vulgare subsp. vulgare*	62.53	17/92	59	6.750
L32	gi|296086060	Unamed protein product	*V. vinifera*	17.29	18/91	234	100
L7	gi|296089720	Unnamed protein product	*V. vinifera*	27.96	7/85	55	0.160
L92	gi|147821099	Hypothetical protein VITISV_038267	*V. vinifer*a	39.24	11/99	66	3.538

MP/SC: Number of matched peptides/sequence coverage percentage; MW: Theoretical molecular weigh;

^a^: Spot number corresponds to the 2-DE gel in Figure 3.

^b^: gi number is from NCBI database of matched protein.

^c^: Ratio means the ratio of Mg-deficiency to control; 0 means protein spots were only detected in control roots; 100 means protein spots were only detected in the Mg-deficient roots.

**Table 2:** **List of differentially expressed proteins identified using MALDI-TOF/TOF-MS in magnesium (Mg)-deficient** ***Citrus sinensis*** **roots**

**Spot no.** ^**a**^	**Accession no.** ^**b**^	**Protein identity**	**Organism**	**MW (kDa)**	**MP/SC**	**Score**	**Ratio** ^**c**^
***Carbohydrate and energy metabolism***							
R85	gi|255579310	Pyruvate decarboxylase, putative	*Ricinus communis*	65.33	11/99	204	0.062
R95	gi|332195235	Phosphoglycerate kinase	*Arabidopsis thaliana*	49.91	17/92	110	0.235
R83	gi|710400	Pyruvate dehydrogenase E1 alpha subunit	*A. thaliana*	43.00	16/93	56	3.440
R38	gi|951369	Ferredoxin NADP reductase	*Pisum sativum*	10.82	8/101	53	2.495
R111	gi|222356610	ATPase alpha subunit, partial (mitochondrion)	*Afrothismia gabonensis*	40.27	15/94	210	2.279
R36	gi|302835814	Adenylate kinase	*Volvox carteri f. nagariensis*	25.89	11/78	60	2.446
R120	gi|33149683	Alcohol dehydrogenase	*Dianthus caryophyllus*	41.23	8/101	287	2.764
R84	gi|327555177	Beta-amylase 8	*Hordeum vulgare subsp. vulgare*	51.37	17/93	57	3.121
R121	gi|11066213	Hexokinase	*Citrus sinensis*	54.02	14/93	253	6.019
***Protein metabolism***							
R1	gi|255584432	Proteasome subunit alpha type, putative	*R. communis*	25.99	11/97	190	100
R11	gi|255620897	Zinc metalloprotease, putative	*R. communis*	17.94	9/99	57	3.011
R13	gi|255543801	Cysteine protease, putative	*R. communis*	41.04	7/103	91	4.924
R35	gi|332006674	Putative S9 tyrosyl aminopeptidase	*A. thaliana*	81.26	12/97	73	100
R57	gi|332006104	Eukaryotic translation initiation factor 3B-2	*A. thaliana*	82.12	16/93	56	2.402
R34	gi|806808	Chaperonin precursor	*P. sativum*	62.95	12/97	131	0.180
R56	gi|806808	Chaperonin precursor	*P. sativum*	62.95	19/90	232	0.439
R2	gi|124484511	Alpha chain of nascent polypeptide associated complex	*Nicotiana benthamiana*	21.91	10/47	215	0.089
***Stress responses***							
R14	gi|23477636	Grp94 (HSP)	*Xerophyta viscosa*	92.90	24/84	240	2.990
R70	gi|227438123	Disease resistance protein	*Brassica rapa subsp. pekinensis*	81.89	19/90	57	3.881
R53	gi|399940	Heat shock 70 kDa protein, mitochondrial		72.49	25/84	330	0.319
R100	gi|4028567	Heat shock protein HSP26	*Triticum aestivum*	26.48	12/97	66	0.325
***Nucleic acid metabolism***							
R37	gi|90403817	RNA polymerase beta chain	*Beaucarnea recurvata*	94.49	22/87	72	0.481
R68	gi|226528292	Spliceosome RNA helicase BAT1	*Zea mays*	45.12	19/91	245	0.456
R82	gi|33945882	Transcription factor homolog BTF3-like protein	*Lotus japonicus*	17.85	11/98	242	0.272
***Cell wall and cytoskeleton metabolism***							
R55	gi|255115691	Actin 1	*Boehmeria nivea*	41.64	19/90	245	5.833
R69	gi|71386188	Villin 3	*Medicago sativa*	20.31	5/104	123	3.134
R101	gi|225454452	Tubulin gamma-1 chain	*Vitis vinifera*	53.25	12/97	104	2.600
***Lipid metabolism***							
R54	gi|1117793	Lipoxygenase	*Solanum tuberosum*	99.60	14/95	65	2.074
R99	gi|870726	Biotin carboxylase subunit	*Nicotiana tabacum*	58.35	28/80	419	0.289
***Other and unknown biological processes***							
R3	gi|30017553	Unknown protein, 5’-partial	*Oryza sativa Japonica Grou*	10.38	5/104	46	2.086
R12	gi|296086893	Unnamed protein product	*Vitis vinifera*	9.09	7/101	55	0.278

MP/SC: Number of matched peptides/sequence coverage percentage; MW: Theoretical molecular weigh;

^a^: Spot number corresponds to the 2-DE gel in Figure 4.

^b^: gi number is from NCBI database of matched protein.

^c^: Ratio means the ratio of Mg-deficiency to control; 0 means protein spots were only detected in control roots; 100 means protein spots were only detected in the Mg-deficient roots.

### Principal component analysis (PCA) loading plots and correlation

**Figure 6:**
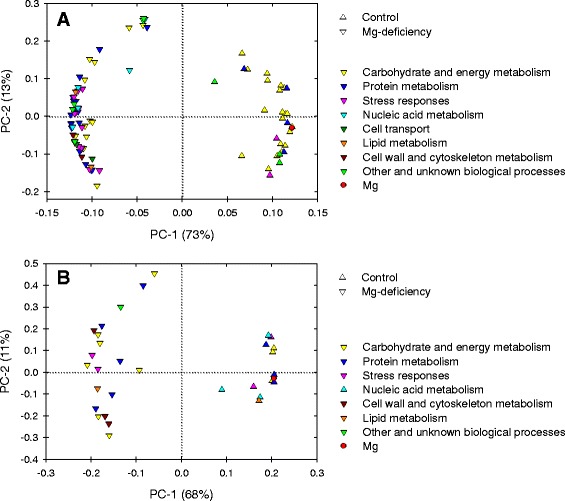
**PCA loading plots of differentially expressed proteins in Mg-deficient leaves (A) and roots (B).**

The majority of differentially expressed proteins involved in protein metabolism and stress responses were highly clustered under Mg-deficient leaves. Furthermore, the differentially expressed proteins associated with nucleic acid metabolism, cell transport, lipid metabolism, and cell wall and cytoskeleton metabolism were only plotted in Mg-deficient leaves (Figure 6A). However, the differentially expressed proteins in nucleic acid metabolism were clustered in control roots. The proteins in cell wall and cytoskeleton metabolism were only plotted in Mg-deficient roots (Figure 6B).

**Figure 7:**
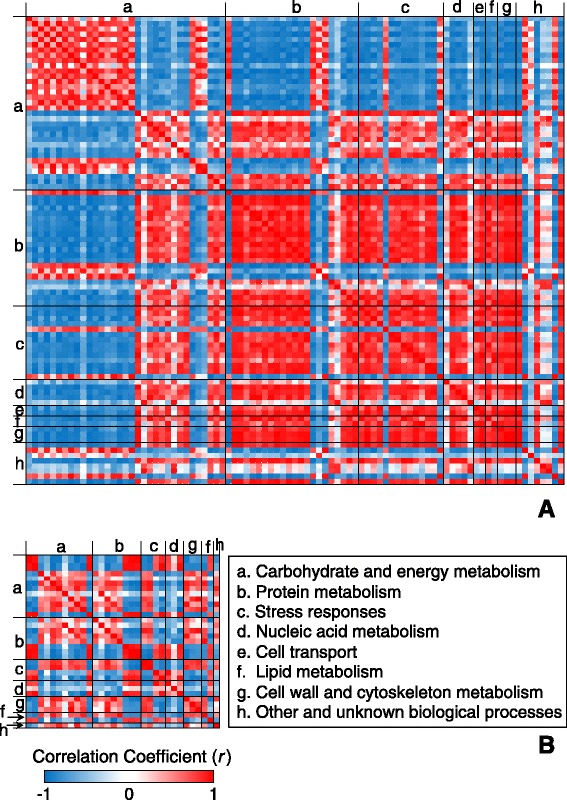
**Pearson correlation coefficient matrix for the differentially expressed protein spots in Mg-deficient leaves (A) and roots (B).** Red and blue colors indicated positive and negative correlation between the differentially expressed proteins.

The correlation coefficient matrix presented that the individual differentially expressed proteins were highly correlated within each categorized metabolism, regardless of tissues (Figure 7). In leaves, the majority of the differentially expressed proteins in carbohydrate and energy metabolism were positively correlated with each other but negatively with the other proteins in the other metabolisms. The most proteins in the other metabolisms but carbohydrate and energy metabolism were highly positively correlated with each other (Figure 7A). In contrast, the differentially expressed proteins in roots did not show any clear pattern like those in leaves (Figure 7B).

### Transcriptional analysis of genes for some differentially expressed proteins

**Figure 8:**
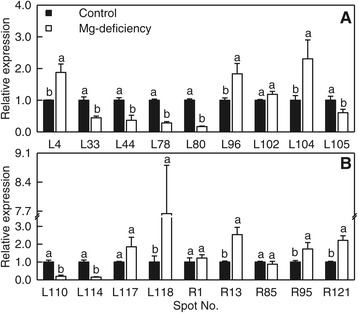
**Relative expression of 13 genes from leaves and of five genes from roots. (A)** Relative expression of nine leaf genes encoding cysteine proteinase (L4); ATP synthase CF1 alpha subunit, chloroplastic (L33), triosephosphate isomerase (L44), Cu/Zn superoxide dismutase (L78), phosphoglycerate kinase (L80), ascorbate peroxidase (L96), S-adenosylmethionine synthetase (L102), aconitate hydratase 3 (L104), nucleoside diphosphate kinase, putative (L105). **(B)** Relative expression of four leaf genes encoding ATYKT62 (L110), glyceraldehyde-3-phosphate dehydrogenase B subunit (L114), alcohol dehydrogenase (L117), and ribulose 1,5-bisphosphate carboxylase, partial (L118), and five root genes encoding proteasome subunit alpha type, putative (R1), cysteine protease, putative (R13), pyruvate decarboxylase, putative (R85), phosphoglycerate kinase (R95), and hexokinase (R121). Bars represent means ± SE (*n* = 3). Different letters above the bars indicate a significant difference at *P* < 0.05. All the values were expressed relative to the control leaves or roots.

To verify the changed expression in transcriptional level and evaluate the correlation between mRNA and protein levels. The expression levels of genes for 18 differentially expressed proteins from Mg-deficient leaves (i.e. L4, 33, 44, 78, 80, 96, 102, 104, 105, 110, 114, 117 and 118) and roots (i.e. R1, 13, 85, 95 and 121) were analyzed by qRT-PCR (Figure 8). Of the 18 genes, the expression profiles of nine genes (i.e. L4, 33, 44, 80, 96, 104 and 114, and R13 and 121) were well correlated with our 2-DE data (Tables 1 and 2), meaning that the differentially expressed proteins are regulated at the transcriptional level. However, the transcript level changes of the remaining nine genes (i.e. L78, 102, 105, 100, 117 and 118, and R1, 85 and 95) did not match the proteomic observations (Tables 1 and 2). Indeed, the transcript levels of genes do not necessarily match the levels of their corresponding proteins, since the abundance of a protein depends not only on transcription rate of the gene expression alone [14,16]. The discrepancy between the expression levels of the nine genes and the abundance of the corresponding protein (Table 2 and Figure 8) suggests that post-translational modifications (PTMs) might influence the abundances of these proteins and alter the positions of these proteins on the gel.

## Discussion

### Mg-deficiency triggers different responses in leaves and roots

Our results showed that the amounts of differentially expressed proteins were much more in the Mg-deficient leaves than in the Mg-deficient roots (Tables 1 and 2; Additional file 1), meaning that the effects of long-term Mg-deficiency on protein profiles of leaves were more pronounced than on those of roots. This agrees with the reports in *A. thaliana* that the root transcriptome was less severely affected by long-term (1 week) Mg-deficiency [13], and that the responses of plants to Mg-deficiency was asynchronized, with a lower number of differentially regulated genes in leaves 4 or 8 h after the removal of Mg from the nutrient solution and in roots 28 h after the removal of Mg [12]. This is also supported by our results that Mg-deficiency decreased leaf concentration of total soluble proteins (Figure 2F), but had no influence on root concentration of total soluble proteins (Figure 2H). As shown in Tables 1 and 2, the majority of the differentially expressed proteins only presented in roots or leaves, only two differentially expressed proteins with the same gi number presented in both roots and leaves [i.e. alcohol dehydrogenase (ADH, gi|33149683), which increased in leaves (L117) and roots (R120) in response to Mg deficiency; and chaperonin precursor (gi|806808), which increased in Mg-deficient leaves (L25) and decreased in Mg-deficient roots (R34 and 56)]. In leaves, the abundances of putative ferredoxin-NADP reductase (FNR, L119) and ATP synthase (L33, 47 and 65) decreased and the levels of proteins involved in TCA cycle (L81, L89 and L104) as well as dark respiration were increased in response to Mg-deficiency; whereas in roots, the levels of FNR (R38) and ATPase α subunit, partial (mitochondrion, R111) were upregulated and the abundance of pyruvate decarboxylase, putative (R85) as well as respiration was downregulated by Mg-deficiency. In conclusion, there are many differences in Mg-deficiency-induced changes in protein profiles as well as biochemical responses between leaves and roots.

As shown in Tables 1 and 2, Mg-deficiency did not alter the abundances of these proteins potentially involved in mediating Mg transport such as the MAGNESIUM⁄PROTON EXCHANGER 1 (MHX1) and the MITOCHONDRIAL RNA SPLICING 2 ⁄MAGNESIUM TRANSPORTER (MRS2 ⁄MGT⁄CorA) family [17]. This agrees with the reports that Mg-deficiency did not induce the expression of genes associated with the transport of Mg, with the only exception of the *MRS2-9* being downregulated [12,13].

Some differentially expressed proteins [i.e. Rubisco activase (L20, 24, 26, 31 and 51), phosphoglycerate kinase (L80, 93 and 94), cysteine proteinase (L4 and 6)] in Mg-deficient leaves and chaperonin precursor (R34 and 56) in Mg-deficient roots were represented by more than one spot (Tables 1 and 2). This might reflect isozymes or PTMs of a single protein [18].

### Proteins involved in carbohydrate and energy metabolism

A crucial role of Mg is its involvement in the export of carbohydrates from source to sink sites. The accumulation of sugars in source leaves, an early symptom of Mg-deficiency, usually precedes the decreases in photosynthetic rate and Chl concentration [2,5,10]. The accumulation of sugars in leaves may repress the expression of genes that encode photosynthetic enzymes, thus decreasing Chl concentration and photosynthetic capacity [19]. Therefore, these proteins involved in photosynthesis and carbohydrate metabolism and related biological processes might be altered under Mg-deficiency. As expected, many differentially expressed proteins related to carbohydrate and energy metabolism were found in Mg-deficient leaves (Table 1 and Additional file 2). Our finding that Mg-deficiency decreased the abundances of Rubisco (L118) and Rubisco activase (L20, 24, 26, 31, 35, 51 and 52) (Table 1) agrees with the report that Mg-deficiency reduced the activities of Rubisco and Rubisco activase and the expression levels of genes encoding Rubisco large subunit (rbcL), Rubisco small subunit (rbcS), and Rubisco activase subunit (rca) in spinach (*Spinacia oleracea*) leaves [20]. Mate et al. [21] reported that transgenic tobacco (*Nicotiana tabacum*) plants expressing an antisense gene encoding Rubisco activase reduced Rubisco carbamylation, hence impairing photosynthesis. Two studies with logan (*Dimocarpus longana* [22] and flowering Chinese cabbage (*Brassica campestris*) [23] revealed that Mg-deficiency-induced inhibition of photosynthesis might be associated with both reduced carboxylation efficiency and lower rate of ribulose-1,5-bisphosphate regeneration. Oxygen evolving enhancer proteins (OEEs) consist of three subunits, OEE 1 (33 kDa), OEE 2 (23 kDa) and OEE 3 (16 kDa). These are nuclear-encoded chloroplast proteins, and peripherally bound to photosystem II (PSII) on the luminal side of the thylakoid membrane. OEE1 is the most important protein for oxygen evolution and PSII stability. Mg-deficiency greatly decreased the level of OEE1 in leaves (L48, Table 1). This means that Mg-deficiency might impair the stability of oxygen evolution and PSII, as found on Mg-deficient *C. sinensis* and *C. grandis* [5]. In addition, Mg-deficient leaves had lower level of photosynthetic electron transfer-like protein (L115, Table 1**)**, which agrees with the report that Mg-deficiency decreased photosynthetic electron transport by impairing the whole photosynthetic electron transport chain from the PSII donor side up to PSI, thus decreasing *Citrus* leaf CO_2_ assimilation [5]. Hajirezaei et al. [24] reported that a small decrease in the activity of FNR by antisense RNA led to decreases in photosynthetic rate and NADPH level, and increases in the extent of Q_A_ reduction and NADP level, and concluded that FNR was one of the rate-limiting steps in photosynthesis and FNR-deficiency-induced inhibition of photosynthesis was caused by impairment of FNR-mediated electron transfer from reduced ferredoxin to NADP. As shown in Table 1, the level of putative FNR (L119) decreased in Mg-deficient leaves. This means that FNR activity might be downregulated by Mg-deficiency, thus impairing the photosynthetic electron transfer and increasing the extent of Q_A_ reduction, and hence inhibiting photosynthesis. Palatnik et al. [25] demonstrated that FNR-deficient plants were particularly prone to photoinhibitory damage and photooxidative injury. All these results agree the report that Mg-deficiency decreased CO_2_ assimilation, and increased the extent of Q_A_ reduction and the concentration of malondialdehyde (MDA) in *Citrus* leaves [5].

The reduction of 3-phosphoglycerate to triose phosphate, which is reversibly catalyzed by two chloroplast enzymes [i.e. NADP-glyceraldehyde-3-phosphate dehydrogenase (GAPDH) and phosphoglycerate kinase (PGK)], is a crucial step in photosynthesis linking the photochemical events of the thylakoid membranes with the carbon metabolism of the photosynthetic carbon-reduction cycle in the stroma. Price et al. [26] showed that antisense transgenic plants with severely reduced chloroplast GAPDH activity were not photoinhibited despite the continuous presence of a large thylakoid proton gradient in the light, and that the electron-transport chain did not become over-reduced due to a shortage of NADP despite the downregulation of photosynthetic electron transport due to the build-up of a large proton gradient. The observed lower level of GAPDH B subunit (L114, Table 1) might be helpful to lessen photoinhibition and the over-reduction of photosynthetic electron transport, thus lowering photooxidative damage caused by Mg-deficiency. In addition, Mg-deficiency decreased leaf abundance of PGK (L80, 93 and 94, Table 1). Therefore, the reduction of 3-phosphoglycerate to triose phosphate might be decreased in Mg-deficient leaves due to lower activities of chloroplast GADPH and PGK.

Mg-deficiency also reduced leaf levels of putative carbonic anhydrase (L106), triosephosphate isomerase (L44), fructose-bisphosphate aldolase, class I (L108) and putative chloroplastic lactoylglutathione lyase (L67) (Table 1). Carbonic anhydrase, which reversibly catalyzes the interconversion of CO_2_ and HCO_3_^−^, is a major protein constituent of the C_3_ higher plant chloroplast. Stimler et al. [27] showed that stomatal response was mediated by carbonic anhydrase, and that H_2_S formed in the mesophyll *via* the reaction of carbonyl sulfide and water with carbonic anhydrase was involved in the stomatal response. The observed lower abundance of carbonic anhydrase means that its activity might be downregulated in Mg-deficient leaves, thus decreasing the stomatal conductance (Figure 2B). However, in addition to stomatal factor, non-stomatal factors such as photosynthetic inhibition also contributed to the lower CO_2_ assimilation in Mg-deficient leaves, as indicated by the increased intercellular concentration (Figure 2C). Ito et al. [28] showed that triosephosphate isomerase and putative plastidic aldolase were inactivated by oxidized glutathione (GSSG) and reactivated by reduced glutathione (GSH). This agrees with the report that Mg-deficient *Citrus* leaves had increased GSSG concentration, but decreased GSH level [5]. Haake et al. [29] reported that a moderate decrease of plastidic aldolase activity led to a decrease in photosynthesis in antisense transgenic potato plants. Lactoylglutathione lyase (also known as glyoxalase I) catalyzes the formation of S-d-lactoylglutathione in the presence of glutathione and methylglyoxal, a cytotoxic compound produced spontaneously under physiological conditions from the glycolysis and photosynthesis intermediates glyceraldehyde-3-phosphate and dihydroxyacetone phosphate. Besides detoxification of methylglyoxal, the glyoxalase system could also play a role in oxidative stress tolerance by recycling GSH and maintaining glutathione homeostasis [30]. The observed lower level of putative chloroplastic lactoylglutathione lyase (L67) in Mg-deficient leaves (Table 1) agrees with our previous report that Mg-deficiency increased GSSG concentration and decreased GSH concentration in *Citrus* leaves [5]. To conclude, Mg-deficiency decreased the levels of many proteins involved in photosynthesis including Rubisco, Rubisco activase, OEE1, photosynthetic electron transfer-like protein, FNR, aldolase, thus decreasing leaf photosynthesis.

We found that Mg-deficiency increased the abundances of NADP-malate dehydrogenase (L81), putative succinate dehydrogenase (SDH, L89) and aconitate hydratase (ACO) 3 (L104) in *C. sinensis* leaves (Table 1), which agrees with our data that Mg-deficient leaves had higher rate of dark respiration (Figure 2E) and the report that the activities of enzymes related to glycolysis and TCA cycle were enhanced in Mg-deficient *C. sinensis* leaves [8]. NADP-malate dehydrogenase is the key enzyme in the malate/oxalacetate shuttle, which is the major machinery for the transport of excess reducing equivalents generated in chloroplasts to mitochondria [31]. NADP-malate dehydrogenase converts oxalacetate to malate using NADPH, facilitating the regeneration of the electron acceptor NADP in the chloroplasts, particularly when CO_2_ assimilation is restricted [32]. The observed higher level of NADP-malate dehydrogenase in Mg-deficient leaves might contribute to the transport of excess reducing equivalents from chloroplasts to mitochondria and the regeneration of chloroplast NADP, thus preventing oxidative damage and enhancing the tolerance of plant to Mg-deficiency. Previous study showed that antisense inhibition of the iron-sulphur subunit of SDH and ACO reduced the carbon flow through the TCA cycle, and enhanced the photosynthesis and stomatal conductance [33]. This agrees with our data that Mg-deficient leaves had increased dark respiration (Figure 2E), levels of SDH and ACO (Table 1), and decreased CO_2_ assimilation (Figure 2A) and stomatal conductance (Figure 2B). Also, the abundances of putative dihydrolipoamide succinyltransferase component of 2-oxoglutarate dehydrogenase (L112) related to TCA cycle, and 2-phospho-D-glycerate hydrolase (L72) and 2,3-bisphosphoglycerate-independent phosphoglycerate mutase 1 (L86) involved in glycolysis in leaves increased in response to Mg-deficiency (Table 1).

Our finding that Mg-deficient leaves had increased abundance of α-amylase (L60, Table 1), a starch degrading enzyme agrees with the report that the activities of two starch degrading enzymes (α-amylase and starch phosphorylase) increased in Mg-deficient spruce needles [34]. Thus, starch degradation might be upregulated in Mg-deficient *C. sinensis* leaves. However, Mg-deficiency had little influence on starch concentration in *C. sinensis* leaves [5]. This might be related to the fact that sucrose biosynthesis and export decreased in response to Mg-deficiency [34]. Also, the upregulation of starch biosynthesis was not ruled out, because the abundance of ADP-glucose pyrophosphorylase catalytic subunit (L77) increased in Mg-deficient leaves. Thus, it is reasonable to assume that the rate of starch turnover in Mg-deficient leaves might be enhanced [34].

Mg-deficient leaves had lower levels of putative ATP synthase β subunit (L61), ATP syntase γ chain 2 (L65), chloroplastic ATP synthase CF1 α subunit (L33) and chloroplastic ATP synthase subunit α (L47) (Table 1). This means that ATP synthesis catalyzed by ATP synthase in Mg-deficient leaves might be decreased, which might provide an advantage to prevent energy surplus due to increased dark respiration and the generation of excess reducing equivalents in chloroplasts.

Adenosine kinase, which catalyzes the reaction: adenosine + ATP → AMP + ADP, is an essential component for maintaining purine nucleotide pools in *Arabidopsis*. It also contributes to cytokinin interconversion in *Arabidopsis* [35] and plays a key role in sustaining transmethylation reactions by serving as a coarse metabolic control to reduce the concentration of free adenosine in spinach and sugar beet (*Beta vulgaris*) cells during salt stress [36]. Our finding that the abundance of putative adenosine kinase (L40) in leaves increased in response to Mg-deficiency (Table 1) agrees with the report that adenosine kinase activity, protein and transcripts in spinach and sugar beet leaves were enhanced by salt stress [36].

Nucleoside diphosphate kinase (NDPK) is a ubiquitous housekeeping enzyme that maintains the intracellular levels of all (d)NTPs used in biosynthesis except for ATP. In plants, NDPK2 is known to regulate the expression of antioxidant genes. Transgenic potato, sweet potato and *Arabidopsis* plants overexpressing *Arabidopsis NDPK2* had increased activities of antioxidant enzymes and enhanced tolerance to methylviologen, salt and temperature stresses [37,38]. Aldehydes, which lead to a rapid and excessive accumulation of ROS in plants, may be induced by various abiotic stresses. Alcohol dehydrogenase (ADH), which converts aldehydes into alcohols, is essential for plants to survive under anaerobic conditions. ADH gene and/or protein are induced by ABA, salt, desiccation, high and low temperatures in various plants. It has been suggested that the induction of ADH by these stresses may trigger a signal transduction cascade, which would lead to a decrease in membrane damage, through adapting plants to oxidative stress [39,40]. Therefore, the antioxidant capacity might be upregulated in Mg-deficient leaves due to the increased level of putative NDPK (L105) and ADH (L117), hence enhancing the Mg-deficiency tolerance. This agrees with our report that Mg-deficient *C. sinensis* leaves had higher activities of ascorbate peroxidase (APX), superoxide dismutase (SOD), glutathione reductase (GR), dehydroascorbate reductase (DHAR) and guaiacol peroxidase (GPX) [5].

Unlike to leaves, Mg-deficiency decreased root respiration (Figure 2G) and the activities of enzymes related to glycolysis and TCA cycle [8]. As expected, Mg-deficiency decreased the abundances of putative pyruvate decarboxylase (R85) and PGK (R95) in roots. However, root level of pyruvate dehydrogenase E1 α subunit (R83) increased in response to Mg-deficiency (Table 2).

In contrast to leaf putative FNR (L119, Table 1), the level of root FNR (R38, Table 2) was increased by Mg-deficiency. In higher plants, there are two forms of FNR, a photosynthetic FNR and a heterotrophic FNR. Onda et al. [41] revealed that the interaction of root FNR with ferredoxin (Fd) is crucial for an efficient electron flux of NADPH-FNR-Fd cascade, thus supporting Fd-dependent metabolism in non-photosynthetic organs. Xu et al. [42] showed that the abundance of a FNR precursor was reduced by 60% in the roots of null transformant (NT) plants, but maintained in transgenic lines expressing the adenine isopentenyl transferase gene under heat stress, and concluded that the maintenance of FNR level under heat stress could reflect a superior N assimilation capacity of the transgenic lines over the NT line that facilitates their growth under heat stress. Thus, the observed higher level of FNR (R38) in Mg-deficient roots (Table 2) might be an adaptive response.

The biosynthesis of ATP in Mg-deficient roots might be enhanced due to increased level of mitochondrial ATPase α subunit, partial (R111, Table 2). Adenylate kinase catalyses the reversible formation of ADP by the transfer of one phosphate group from ATP to AMP, thus equilibrating adenylates [43]. Thus, the increased abundances of the two proteins might be advantage to maintaining energy balance in Mg-deficient roots, when ATP production was reduced due to decreased respiration (Figure 2G). Like leaves, the level of root ADH (R120) increased in response to Mg-deficiency. Similar result has been obtained on B-deficient *C. sinensis* roots [16].

Similar to leaf α-amylase, the activity of β-amylase might be upregulated in Mg-deficient roots due to enhanced abundance of β-amylase 8 (R84, Table 2), thus increasing the breakdown of starch into sugars in roots. This agrees with our report that Mg-deficiency decreased the concentration of starch in *Citrus* roots [5].

Hexokinases (HXKs) phosphorylate glucose to produce glucose-6-phosphate, the first step of glycolysis. Our finding that Mg-deficient roots had higher level of HXK (R121) compared with controls (Table 2) agrees with the reports that HXKs contributed to the survival of acclimated maize root tips *via* hypoxic pretreatment by allowing the maintenance of a sustained glycolytic rate [44], and that OsHXK7 (*Oryza sativa* hexokinase 7), localized at the cytosol, was upregulated under sugar-starvation conditions but downregulated in response to high sugar treatments of the leaf tissues of rice [45], because Mg-deficiency decreased or did not affect the concentrations of non-structural carbohydrates in *C. sinensis* roots [5]. Also, HXKs might act as a glucose sensor (regulatory function) and, through its catalytic activity, as a crucial regulator of ROS levels in plant organelles such as mitochondria and chloroplasts [46].

### Proteins involved in stress responses

Because leaf CO_2_ assimilation decreased in response to Mg-deficiency (Figure 2A), less of the absorbed light energy was utilized in photosynthetic electron transport, particularly under high light. The excess absorbed photon flux can potentially lead to ROS production. Thus, ROS production might be enhanced in Mg-deficient leaves [5]. To minimize the cellular damage caused by ROS, plants have evolved an antioxidant system composed of antioxidants and antioxidant enzymes. As expected, the leaf levels of antioxidant enzymes (L50, 63, 78 and 96) increased in response to Mg-deficiency except for chloroplast Cu/Zn SOD (L97) (Table 1**)**. This agrees with the report that Mg-deficient *Citrus* leaves had higher or similar activities of antioxidant enxymes [APX, monodehydroascorbate reductase, DHAR, GR, SOD and GPX] except for catalase [5]. Aldo-keto reductases (AKR), which catalyze the reduction of aldehyde to alcohol, are effective detoxification of peroxidation-derived reactive aldehydes [47]. Transgenic tobacco plants overexpressing alfalfa or rice *ARK* had enhanced tolerance against a variety of oxidative damages induced by methylviologen, UV-B irradiation, heavy metals, drought, heat treatment, osmotic and salt stresses [47-49]. The upregulation of the putative AKR (L74 and 103) in Mg-deficient leaves (Table 2) might contribute to the oxidative tolerance of leaves. In addition, Mg-deficiency increased the leaf abundances of heat shock proteins (HSPs, L28, 39, 46 and 59, Table 1), which play a key role in protecting plants against stress by reestablishing normal protein conformation and thus cellular homeostasis, and stress-related protein (L76 and 90, Table 1). Based on these results, we concluded that proteins associated with stress-response functions were upregulated in Mg-deficient leaves. However, the level of late embryogenesis abundant (LEA) protein (L116) in leaves decreased in response to Mg-deficiency (Table 1).

Like to leaves, the levels of stress-responsive proteins [Grp94 (HSP, R14) and disease resistance protein (R70)] in roots increased in response to Mg-deficiency (Table 2). This agrees with the reports that B-deficiency increased the level of NB-ARC domain-containing disease resistance protein in *C. sinensis* roots [16], and that the abundance of Grp94 in *Xerophyta viscosa* plants increased in response to heat and dehydration [50]. However, the abundances of mitochondrial heat shock 70 kDa protein (R53) and heat shock protein HSP26 (R100) were decreased in Mg-deficient roots (Table 2), which agrees with the studies that B-deficiency decreased the levels of several HSPs (HSP70, HSP70 family proteins, mitochondrial HSO70 2 and HSP10) in *C. sinensis* roots [16] and the abundances of three HSPs in *Lupinus albus* roots [51].

### Proteins involved in protein metabolism

Protein synthesis elongation factor Tu (EF-Tu) plays a central role in the elongation phase of protein synthesis in bacteria and organelles including mitochondria and plastids in plants [52]. We found that the abundance of the putative enongation factor tu (L79) in leaves decreased in response to Mg-deficiency (Table 1), meaning that the biosynthesis of some proteins in plastids and mitochondria might be downregulated. This agrees with our results that the levels of many chloroplastic (L118, 20, 26, 31, 51, 52, 24, 48, 115, 33 and 67) proteins was decreased by Mg-deficiency (Table 1), and that the concentration of total soluble proteins in leaves decreased in response to Mg-deficiency (Figure 2G). However, the levels of chloroplastic chaperonin precursor (L25), chloroplastic small ribosomal protein 4, partial (L71) and ribosomal protein S3 (L88) related to protein folding and biosynthesis were increased in Mg-deficient leaves (Table 1).

Plant proteases play key roles in maintaining strict protein quality control and degrading specific sets of proteins in response to environmental and developmental stimuli. The majority of the thousand or more proteins that are present in mitochondria are required to be imported from nuclear-encoded cytosolically synthesized precursors, and a number of peptidases are needed to remove the “transient” targeting information present on many, but not all, mitochondrial precursor proteins [53]. The upregulation of mitochondrial processing peptidase β subunit (L109) in Mg-deficient leaves (Table 1) agreed with the increased requirement for removing the “transient” targeting information due to enhanced import resulting from the increased abundance of the putative mitochondrial import inner membrane translocase subunit Tim17/Tim22/Tim23 family protein (L87, Table 1). Ubiquitination, which serves as a versatile PTM, plays a key role in regulating plant response to abiotic stresses. It has been known that the F-box is a motif for ubiquitin dependent proteolysis in cell cycle regulation and signal transduction [54], and that the RING zinc-finger domain is essential for the function of ubiquitin E3 ligases [55]. The upregulation of F-box domain-containing protein (L41), putative F-box family protein, expressed (L8), putative F-box/kelch-repeat protein (L9) and putative RING zinc finger protein (L62) in Mg-deficient leaves (Table 2) means that leaf ubiquitination might be enhanced in response to Mg-deficiency. Also, the abundances of proteins (L4, 6, 45, 49 and 58) related to protein degradation increased in Mg-deficient leaves (Table 1). In conclusion, proteolysis might be enhanced in Mg-deficient leaves, thus lowering leaf concentration of total soluble proteins (Figure 2F). However, the abundances of leaf F2E2.12 (membrane-anchored ubiquitin-fold protein 6 precursor, L27) and putative skp1 protein [an essential component of the SCF (SKP1-CUL1-F-box protein) ubiquitin ligase complex, L98] decreased in response to Mg-deficiency (Table 1).

Amino acids are the structural units that make up proteins and also serve as precursors for many metabolites with multiple functions in plant growth and response to various stresses. All the four differentially expressed proteins (L75, 91, 102 and 113) involved in amino acid metabolism were upregulated in Mg-deficient leaves (Table 1), meaning that the metabolism of amino acids might be upregulated in Mg-deficient leaves. Glutathione S-transferases (GSTs), which catalyze the conjugation of cytotoxic metabolites with the GSH, are mainly involved in detoxifying oxidative-stress metabolites. Glutamine synthetase (GS) catalyses the critical incorporation of inorganic ammonium into the amino acid glutamine. In plants, two types of GS isozymes, located in the cytosol (GS1) and in the chloroplast (GS2) have been found. Transgenic plants overexpressing *GS1* or *GS2* displayed enhanced tolerance to water or salt stress, respectively [56,57]. Methylation induced by abiotic stress has been supposed to be linked with the numerous biochemical pathways involved in acclimatization and stress response in plants [58]. S-adenosylmethionine is an important methyl donor for numerous transmethylation reactions. S-adenosylmethionine synthase is a crucial enzyme that directs the flux of methione to S-adenosylmethionine. These results indicate that the upregulation of amino acid metabolism in Mg-deficient leaves might be involved in the adaption of Mg-deficient plants.

Proteolytic cleavage of proteins by proteases is not limited to the total degradation of mature proteins to free amino acids, but also is relevant for modification and maturation of proteins. Like to leaves, Mg-deficiency increased the abundances of root proteins (R1, 11, 13 and R35) involved in proteolysis (Table 2). However, there was no significant difference in total soluble protein concentration between Mg-deficient and control roots (Figure 2H). This implies that the Mg-deficiency-induced increase in protease levels may be mainly involved in modification and maturation of proteins or that the biosynthesis is enhanced in Mg-deficient roots. The upregulation of proteases in Mg-deficient roots might be an adaptive response of plants to Mg-deficiency through maintaining the protein complexes and/or the recycling of N.

The eukaryotic translation initiation factor 3 (eIF3) has multiple roles during the initiation of translation of cytoplasmic mRNAs. The classic functions ascribed to eIF3 include: (*a*) facilitating the charging of the 40S ribosomal subunit with the ternary complex; (*b*) bridging between the 40S ribosomal subunit and the eIF4G subunit of the cap-binding complex, eIF4F; and (*c*) inhibiting the association of 40S and 60S ribosomal subunits [59]. The upregulation of eIF 3B-2 (R57) in Mg-deficient roots (Table 2) might contribute to the translatability of cytoplasmic mRNAs, thus maintaining the level of cytoplasmic proteins, which agrees with our result that Mg-deficiency did not significantly affect root concentration of total soluble proteins (Figure 2F). However, the levels of root proteins (R2, 34 and 56) involved in protein folding and biosynthesis were decreased by Mg-deficiency.

### Proteins involved in nucleic acid metabolism

The downregulation of RNA polymerase β chain (R37), spliceosome RNA helicase BAT1 (R68) and transcription factor homolog BTF3-like protein (R82) in Mg-deficient roots (Table 2) agrees with our report that all 60 differentially expressed proteins related to nucleic acid metabolism were reduced in B-deficient *C. sinensis* roots except for argonaute family protein [16]. This indicates that the synthesis of RNA might be inhibited in Mg-deficient roots. By contrast, the abundances of all the four altered proteins (L5, 17, 22 and 73) related to nucleic acid metabolism in leaves increased in response to Mg-deficiency (Table 1), meaning that the metabolism of nucleic acid might be upregulated in Mg-deficient leaves.

DEAD box RNA helicases are prominent candidates for RNA chaperones because these proteins can use energy derived from ATP hydrolysis to actively disrupt misfolded RNA structures so that correct folding can occur, and play important roles in plant stress responses [60,61]. Transgenic *Arabidopsis* plants overexpressing a rice gene *OsBIRH1*, which encodes a DEAD-box RNA helicase protein, showed enhanced disease resistance against *Alternaria brassicicola* and *Pseudomonas syringae*, and increased tolerance to oxidative stress and elevated expression levels of oxidative defense genes [61]. The upregulation of DEAD box RNA helicases (L73) in Mg-deficient leaves (Table 1) might be an adaptive response of plants to Mg-deficiency. In nonplant systems, the splicing of group-II introns is mediated by proteins encoded within the introns themselves (known as “maturases”), whereas only a single maturase ORF (*matR*) has retained in the mitochondrial genomes in plants. Interestingly, higher plant genomes contain four maturase-related genes, which exist in the nucleus as self-standing ORFs. Recently, Keren et al. [62] showed that AtnMat2, a nuclear-encoded maturase, was required for splicing of group-II introns in *Arabidopsis* mitochondria. In another study, Keren et al.[63] observed that nMAT1 functions were required for mitochondrial biogenesis and that *nMat1* mutants displayed growth and developmental defect phenotypes and accumulated high levels of ROS, concluding that nMAT1 was essential for mitochondrial complex I assembly and function. Thus, the observed higher level of maturase, partial (L5, Table 1) in Mg-deficient leaves might be an adaptive response.

### Proteins involved in cell wall and cytoskeleton metabolism

Ca, a constituent of the cell wall, plays a key role in determining the structural rigidity of the cell wall. Also, Ca is a regulator that can exert multiple effects on the structure and dynamics of the actin cytoskeleton [64]. It has been well known that high rhizosphere concentration of Mg, relative to Ca, is inhibitory to the absorption of Ca and *vice versa*. Therefore, the concentration of Ca in Mg-deficient plant tissues should be enhanced. Indeed, Mg-deficiency increased the concentration of Ca in rough-lemon (*Citrus volkameriana*) leaves [65]. Based on these results, it is reasonable to assume that the Mg-deficiency-induced increase in Ca concentration might be responsible for the enhanced abundances of myosin class 11–1 (L16), UGT1 (UDP-glucosyltransferase, L21) and pollen coat oleosin-glycine rich protein (L107) involved in cell wall and cytoskeleton metabolism in Mg-deficient leaves (Table 1). Similarly, the abundances of root actin 1 (R55), villin 3 (R69) and tubulin γ-1 chain (R101) were upregulated by Mg-deficiency (Table 2), which is in agreements with the report that Mg-deficiency led to enhanced suberization in endodermis and hypodermis of corn roots [66]. The upregulation of proteins related to cell wall and cytoskeleton metabolism in Mg-deficient roots and leaves might be an adaptive response of plants to Mg-deficiency.

### Proteins involved in cell transport

Proteins that reside in endomembrane organelles, or that are secreted from the cell, reach their sites of function by transport of the endomembrane system [67]. The translocase of the inner membrane (TIM) is a complex of proteins found in the inner membrane of the mitochondria and plays a pivotal role in protein import, which is important to cope with stress [53]. The observed higher level of the putative mitochondrial import inner membrane translocase subunit Tim17/Tim22/Tim23 family protein (L87) in Mg-deficient leaves (Table 1) can be explained by the fact that mitochondria are the center of oxygen sensing. Under Mg-deficiency, high protein import might be required for the increased ROS detoxification [5], thus requiring high activity of the TIM protein complex [68]. SNAREs are core proteins for vesicle fusion and can be categorized into two types: SNAREs on the vesicle are called v-SNAREs, while SNAREs on the target membrane are called t-SNAREs. AtYKT61 and AtYKT62, two t-SNARES, are essential for membrane fusion mediated by either SYP41 or STP62 [67,69]. The upregulation of the two proteins means that the transport of proteins might be enhanced in Mg-deficient roots.

### Proteins involved in lipid metabolism

Sato-Izawa et al. [70] revealed that the inositol-1,4,5-trisphosphate 5-phosphatase, an enzyme probably involved in phosphoinositide signaling, was required for many essential cellular functions, such as cytoskeleton organization, endocytosis and vesicular trafficking in eukaryotes. Perera et al. [71] showed that transgenic *Arabidopsis* plants expressing the type 1 inositol-1,4,5-trisphosphate 5-phosphatase had enhanced drought tolerance and altered ABA signaling. Cytochrome P450s (CytoP450s), a superfamily of ubiquitous heme-containing mixed-function monooxygenases proteins, play a key role in biotic and abiotic stresses [72]. In *Arabidopsis*, at least five of the 29 *CytoP450s* are induced by abiotic and biotic stresses including *Alternaria brassicicola* or *Alternaria alternata*, paraquat, rose bengal, UV-C stress, heavy metal stress (CuSO_4_), mechanical wounding, drought, high salinity, low temperature or hormones [73]. Transgenic tobacco and potato plants expressing *CytP450* with increased monooxygenase activity tolerated better oxidative stress after herbicide treatment [72]. Therefore, the observed higher level of putative type 1 inositol-1,4,5-trisphosphate 5-phosphatase CVP2 (L43, Table 1) and putative CytoP450 (L19, Table 1) might contribute to Mg-deficiency tolerance of plants.

We found that the abundance of root lipoxygenase (LOX, R54) increased in response to Mg-deficiency (Table 2), which is in agreement with the report that the level of LOX 2 was enhanced in B-deficient *C. sinensis* roots [16]. LOX, which catalyses the oxidation of α-linolenic acid into either 9- or 13-hydroperoxy-octadecatrienoic acids, or a mixture of both, is one of the key enzymes responsible for the biosynthesis of jasmonates, which are ubiquitously occurring lipid-derived compounds with signal functions in plant responses to abiotic and biotic stresses [74]. Hu et al. [75] demonstrated that *TomloxD*, a tomato 13-LOX gene, was involved in endogenous jasmonate synthesis and tolerance to biotic and abiotic stress. Therefore, both jasmonate biosynthesis and level might be increased in Mg-deficient roots, thus enhancing plant Mg-deficiency tolerance. This inference is also supported by the observation that the expression levels of two genes encoding MYC2, which is a jasmonate-dependent transcription factor and LOX2, which is an enzyme involved in jasmonate biosynthesis [76,77] in roots increased in response to Mg-deficiency (Additional file 2). Thus, jasmonate signaling might be involved in the responses of *Citrus* roots to Mg-deficiency. However, the expression of the gene encoding ZIM/tify-domain (JAZ/TIFY), which is the co-repressor of MYC2 that is ubiquitinilated upon activation of jasmonate signaling cascade [78], did not change in Mg-deficient citrus roots (Additional file 2).

The plastid acetyl-coenzyme A carboxylase, which catalyzes the first committed step of fatty acid synthesis, is present as a heteromeric complex of at least four different protein subunits: the biotin carboxylase, the biotin carboxyl carrier protein, and the α and β subunits of the carboxyltransferase in most plants. Transgenic tobacco plants with less than 25% of wild-type biotin carboxylase levels displayed severely retarded growth when grown under low-light conditions and a 26% lower leaf fatty acid concentration than wild-type plants [79]. Thus, the biosynthesis of fatty acids in Mg-deficient roots might be elevated due to enhanced level of biotin carboxylase subunit (R99, Table 2).

### Other proteins

*Ent*-kaurene is a tetracyclic hydrocarbon precursor for gibberellins (GAs) in plants. In higher plants, *ent*-kaurene is synthesized successively by copalyl diphosphate synthase (CPS) and *ent*-kaurene synthase (KS) from geranylgeranyl diphosphate (GGDP). A range of GA-deficient phenotypes of the *ga1-3* and *ga2-1 A. thaliana* mutants (defective in CPS and KS, respectively) were restored to wild type, when fungal CPS/KS was overexpressed and targeted to plastids. The over-expressing plants emitted *ent*-kaurene into the headspace besides its accumulation in the plant body [80]. Fleet et al. [81] showed that transgenic *Arabidopsis* plants overexpressing *AtCPS* and *AtKS* led to increased *ent*-kaurene production but unchanged concentration of active GAs. Therefore, the upregulation of *ent*-kaurene synthase (L10) in Mg-deficient leaves (Table 1) does not necessarily imply that leaf concentration of GAs was enhanced by Mg-deficiency.

## Conclusions

**Figure 9:**
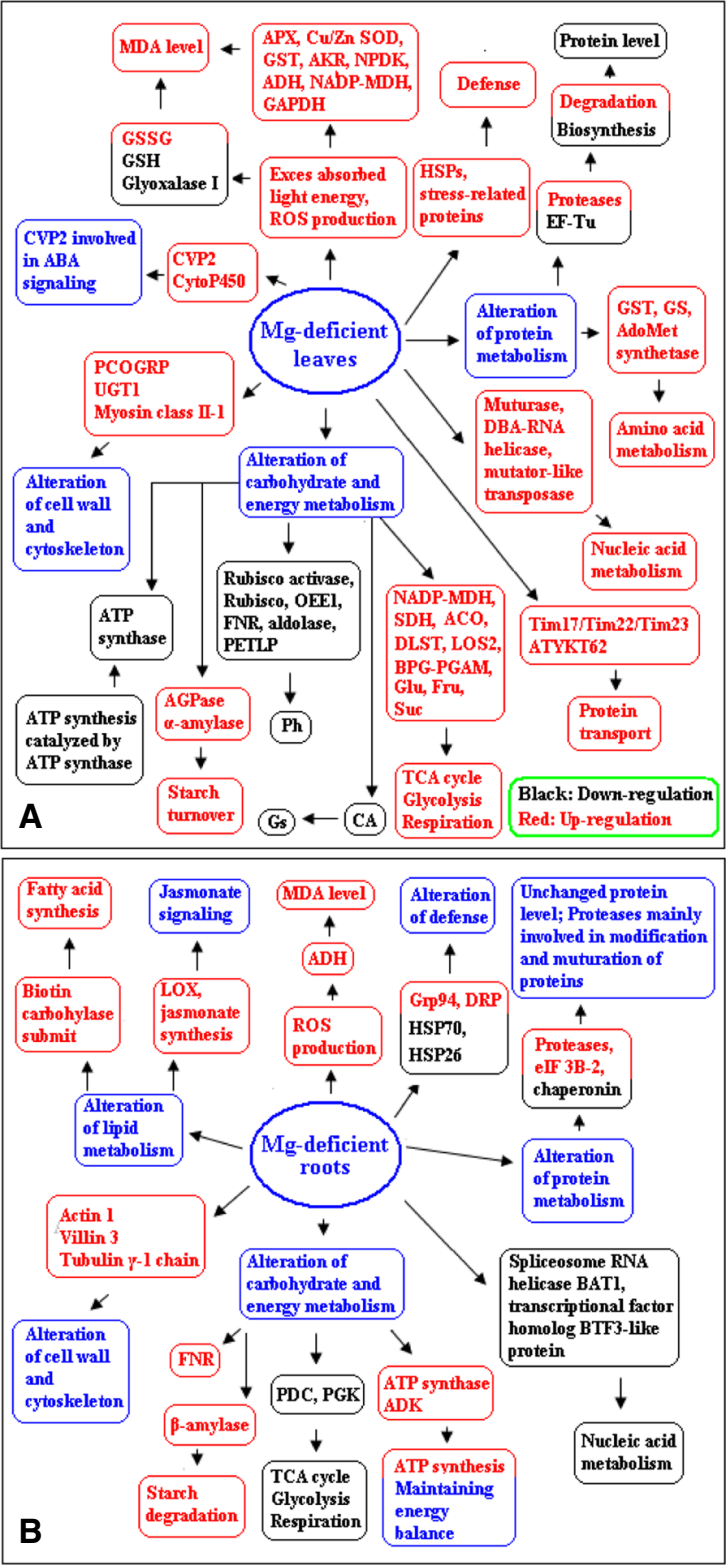
**The potential regulatory network of Mg-deficiency-induced responses in** ***Citrus*** **leaves (A) and roots (B).** BPG-PGAM: 2,3-Bisphosphoglycerate-independent phosphoglycerate mutase 1; CVP2: Type I inositol-1,4,5-trisphosphate 5-phosphatase CVP2; DBA- RNA helicase: Dead box ATP-dependent RNA helicase; DLST: Dihydrolipoamide succinyltransferase component of 2-oxoglutarate dehydrogenase; DRP: Disease resistance protein; Fru: Fructose; Glu: Glucose; Gs: Stomatal conductance; LOS2: 2-Phospho-D-glycerate hydrolase; NADP-MDH: NADP-malate dehydrogenase; PCOGRP: Pollen coat oleosin-glycine rich protein; PDC: Pyruvate decarboxylase; PETLP: Photosynthetic electron transfer-like protein; Pn: Photosynthesis; Suc: Sucrose; Tim17/Tim22/Tim23: Mitochondrial import inner membrane translocase subunit Tim17/Tim22/Tim23 family protein.

We first investigated the proteomic changes induced by long-term Mg-deficiency in *C. sinensis* leaves and roots using 2-DE. In Mg-deficient leaves, 59 upregulated and 31 downregulated proteins were isolated, while only 19 upregulated and 12 downregulated proteins in Mg-deficient roots. This indicated that proteomes were more affected by long-term Mg-deficiency in the leaves than in the roots, which was further supported by our observation that the concentration of total soluble proteins was decreased by Mg-deficiency in leaves, but unaffected in roots. A potential regulatory network of Mg-deficiency-induced responses in *Citrus* leaves and roots was proposed through the integration of the present results and available data in the literatures (Figure 9). Mg-deficiency led to decreased abundances of proteins (Rubisco, Rubisco activase, OEE1, photosynthetic electron transfer-like protein, FNR etc.) involved in photosyntheis, thus decreasing leaf CO_2_ assimilation. The adaptive responses of *C. sinensis* roots and leaves to Mg-deficiency might including several aspects: (*a*) improving leaf respiration and lowering root respiration, but increasing (decreasing) the levels of proteins related to ATP synthase in roots (leaves); (*b*) enhancing the levels of proteins (such as APX, Cu/Zn SOD, GST, AKR, NPDK and ADH) involved in ROS scavenging and other stress-responsive proteins (i.e. HSPs and stress-related proteins); (*c*) accelerating proteolytic cleavage of proteins by proteases, protein transport and amino acid metabolism; and (*d*) upregulating the levels of proteins involved in cell wall and cytoskeleton metabolism. Therefore, our proteomic analysis provides an integrated view of the adaptive responses occurring in Mg-deficient leaves and roots of *C. sinensis*. As a first attempt, the present study will be useful for further investigating the roles of Mg in higher plants. It is worth noting that it may provide more data on Mg-deficiency in real *Citrus* orchards if we use grafted plants rather than *C. sinensis* seedlings as experimental materials, but it is difficult for us to compare the present data with the transcriptomic data obtained on *Arabidopsis* roots and leaves [12,13] and the physiological and biochemical data obtained on *C. sinensis* roots and leaves [5,8]. In the further study, we will investigate the effects of rootstocks on Mg-deficiency-responsive proteomics using grafted citrus plants from different rootstock-scion combinations including both own-rooted scions and rootstocks as controls to obtain more knowledge on Mg-deficiency in real citrus orchards.

## Methods

### Plant culture, Mg treatments and sampling

The study was conducted from February to December, 2011 at Fujian Agriculture and Forestry University. Plant culture, Mg treatments, and sampling were performed according to Yang et al. [5]. Briefly, five-week-old seedlings of ‘Xuegan’ [*Citrus sinensis* (L.) Osbeck] were transplanted to 6 L pots containing sand. Seedlings, two per pot, were grown outdoors at Fujian Agriculture and Forestry University. Each pot was supplied with 500 mL of nutrient solution every other day. The nutrient solution contained the following macronutrients (in mM): KNO_3_, 2.5; Ca(NO_3_)_2_, 2.5; KH_2_PO_4_, 0.5; MgSO_4_, 1; micronutrients (in μM): H_3_BO_3_, 10; MnCl_2_, 2; ZnSO_4_, 2; CuSO_4_, 0.5; (NH_4_)_6_Mo_7_O_24_, 0.065; and Fe-EDTA, 20. Ten weeks after transplanting, each pot was supplied every other day until saturated with Mg-deficient (0 mM MgSO_4_) or Mg-sufficient (1 mM MgSO_4_) nutrient solution for 16 weeks. Sulfur (S) concentration was maintained at a constant level by adding equivalent moles of Na_2_SO_4_ in replace of MgSO_4._ At the end of the experiment, fully-expanded (about 7 weeks old) leaves from different replicates and treatments were used for all the measurements. Leaf discs (0.608 cm^2^ in size) were collected at noon under full sun and immediately frozen in liquid N_2_. Approximately 5-mm-long root apices were frozen immediately in liquid N_2_ after they were excised from the same seedlings used for sampling leaves. Both leaf and root samples were stored at −80°C until extraction.

### Leaf, stem and root DW, and Mg concentration in leaves, stems and roots

At the end of the experiment, 9–10 plants per treatment from different pots were harvested. The plants were divided into leaves, stems and roots. The plant material was then dried at 70°C for 48 h and the DW measured. Mg concentration in leaves, stems and roots was determined by atomic absorption spectroscopy after digested with 1 N HCl [82].

### Total soluble protein concentration in leaves and roots

Total soluble proteins in leaves and roots were extracted with 50 mM Na_2_HPO_4_-KH_2_PO_4_ (pH 7.0) and 5% (w/v) insoluble polyvinylpyrrolidone (PVPP), and measured according to Bradford [83] using bovine serum albumin (BSA) as standard.

### Leaf gas exchange and root and leaf respiration

Measurements of leaf gas exchange were made with a CIRAS-2 portable photosynthesis system (PP systems, Herts, UK) at ambient CO_2_ concentration under a controlled light intensity of 1000 μmol m^−2^ s^−1^ between 10:00 and 11:00 on a clear day. During measurements, leaf temperature and vapor pressure deficit were 30.7 ± 0.1°C and 2.2 ± 0.1 kPa, respectively. Dark respiration was measured with a CIRAS-2 portable photosynthesis system on both control and Mg-deficient leaves at ambient CO_2_ concentration and an ambient temperature at darkness at gas exchange measurements.

Root respiration (O_2_ consumption at 25°C) was measured using Oxy-Lab system from Hansatech (Norfolk, UK) [84].

### Protein extraction for 2-DE

Proteins were extracted from frozen roots and leaves using a phenol extraction procedure [16]. Briefly, about 1 g frozen samples were well ground in liquid N_2_ with a mortar and pestle. Four milliliter of ice-cooled buffer containing 100 mM Tris–HCl pH 7.8, 100 mM KCl, 50 mM L-ascorbic acid, 1% (v/v) Triton X-100, 1% (v/v) β-mercaptoethanol, and 1 mM phenylmethylsulfonyl fluoride was added to the powder and gently pulverized. The mixture was allowed to thaw slowly on ice and then gently grounded for several minutes. The resulting suspension was transferred to a 10 mL tube, then an equal volume of Tris-phenol (pH 8.0) was added. Before centrifuging at 13,000 *g* for 15 min at 4°C, the mixture was thoroughly vortexed. The upper phenolic phase was transferred to a 50 mL Corning tube, then five volumes (about 20 ml) of 100 mM ammonium acetate/methanol was added. After being blended upside down carefully, the mixture was stored at −20°C overnight. The supernatant was removed carefully after centrifugation at 13,000 *g* for 15 min at 4°C, then the protein pellets were resuspended in 25 mL of ice-cooled methanol for 2 h at −20°C. Protein pellets were collected by centrifugation at 13,000 g for 15 min at 4°C, and then were resuspended in 25 mL of ice-cooled acetone containing 0.1% β-mercaptoethanol and kept at −20°C for 2 h. After centrifugation at 13,000 *g* for 15 min at 4°C, the pellets were washed twice with 25 mL of ice-cooled acetone, and then dried by lyophilization and solubilized in lysis buffer [8 M urea, 2 M thiourea, 4% (w/v) 3-[(3-cholamidopropyl) dimethylammonio]-1- propanesulfonate (CHAPS), 2% (v/v) pharmalyte pH 3-10, and 20 mM dithiothreitol (DTT)]. Sample protein concentration was assayed according to Bradford [83]. Three independent protein samples were extracted from three biological replicates for 2-DE.

### 2-DE and image analysis

Protein samples (1.5 mg) were diluted with rehydration solution [8 M urea, 2 M thiourea, 4% (w/v) CHAPS, 0.5% (v/v) IPG buffer, 13 mM DTT, 0.002% (w/v) bromophenol blue] to the total volume of 450 μL and then applied to 24 cm Immobiline DryStrips with a linear pH gradient of 4–7 (GE Healthcare, Uppsala, Sweden) on the rehydration cassette overnight. The isoelectric focusing electrophoresis was carried out using a Ettan IPGphor (GE Healthcare, Uppsala, Sweden) at 200 V for 1.5 h, 500 V for 1.5 h, 1,000 V for 2 h, 0.5 h to increase the voltage from 1,000 V to 8,000 V, 8,000 V for 5 h, 2 h to increase the voltage from 8,000 V to 10,000 V and then 1 h to decrease to 2,000 V until next step. Prior to sodium dodecyl sulphate-polyacrylamide gel electrophoresis (SDS-PAGE), the IPGstrips were subjected to 2 × 20 min equilibration in a buffer solution containing 6 M urea, 50 mM Tris–HCl pH 8.8, 30% (v/v) glycerol and 2% SDS, with a addition of 2% (w/v) DTT in the first equilibration step and 2.5% iodoacetamide in the second step, respectively. The SDS-PAGE was performed with 12% slab gels in running buffer (20 mM Tris–HCl, 192 mM glycine, 0.1% SDS, pH 8.3) at a constant temperature of 15°C by using the Ettan DALTsix Electrophoresis System (GE Healthcare, Uppsala, Sweden) for 6 h at 230 V. SDS-PAGE protein marker (Bio-Rad, Hercules, CA) and liner pH arrangement of IPG strips were used to indicate the pI and molecular mass of the proteins. The 2-D gels were stained by colloidal Coomassie Brilliant Blue G-250 [85]. Briefly, gels were fixed in a solution of 40% (v/v) methanol and 10% (v/v) acetic acid for 40 min, washed with purified water for 3 × 15 min, stained overnight in a staining solution [0.12% Coomassie Brilliant Blue G-250, 20% (v/v) methanol, 10% (v/v) phosphate acid, 10% (w/v) (NH_4_)_2_SO_4_], and then destained with purified water until the background was clear. All these staining steps were conducted on the shaker at room temperature.

Gel images were acquired using Epson Scanner (Seiko Epson Corporation, Japan) at 300 dpi resolution. Image analysis was performed with PDQuest version 8.0.1 (Bio-Rad, Hercules, CA, USA). The software was used to perform background subtraction, Gaussian fitting, gel alignment, spot detection, matching and normalization. The parameters used to spot detection were as follow: sensitivity 6.05, size scale 3, min peak 600, and local regression model was selected to conduct spot normalization. After manual processing, the candidate spots in all triplicate gels were submitted to *t*-test analysis. There was no log transform (or other suitable transform) of the intensities taken prior to analysis when PDquest software was used to process the spot data. The spots, which had both 2-fold cut-off and a *P*-value of less than 0.05 by statistical test were considered differentially expressed protein spots.

### Protein identification by MS/MS and bioinformatic analysis of proteins

Spots were excised from the colloidal Coomassie Brilliant Blue stained gels and plated into a 96-well microtitre plate. Excised spots were firstly destained twice with 60 μL of 50 mM NH_4_HCO_3_ and 50% (v/v) acetonitrile and then dried twice with 60 μL of acetonitrile. Afterwards, the dried pieces of gels were incubated in ice-cold digestion solution (trypsin 12.5 ng/μL and 20 mM NH_4_HCO_3_) for 20 min and then transferred into a 37°C incubator for digestion overnight. Peptides in the supernatant were collected after extraction twice with 60 μL extract solution [5% (v/v) formic acid in 50% (v/v) acetonitrile]. The resulting peptide solution was dried under the protection of N_2_. Before MS/MS analysis, the pellet was redissolved in 0.8 μL matrix solution [5 mg/mL α-cyano-4-hydroxy-cinnamic acid diluted in 0.1% trifluoroacetic acid (TFA), 50% (v/v) acetonitrile]. Then the mixture was spotted onto a MALDI target plate (AB SCIEX). MS analysis of peptide was performed on an AB SCIEX 5800 TOF/TOF. The UV laser was operated at a 400 Hz repetition rate with wavelength of 355 nm. The accelerated voltage was operated at 20 kV, and mass resolution was maximized at 1600 Da. Myoglobin digested with trypsin was used to calibrate the mass instrument with internal calibration mode. All acquired spectra of samples were processed using TOF/TOF Explorer™ Software (AB SCIEX) in a default mode. The data were searched by GPS Explorer (Version 3.6) with the search engine MASCOT (Version 2.3, Matrix Science Inc, Boston, MA). The search parameters were as follow: viridiplantae database (1850050 sequences; 642453415 residues), trypsin digest with one missing cleavage, MS tolerance was set at 100 ppm, MS/MS tolerance was set at 0.6 Da. Protein with ion scores greater than 75 were considered statistically significant (*P* < 0.05) and accepted. Ions score is calculated as −10 × Log(*P*), where *P* is the probability that the observed match is a random event.

Bioinformatic analysis of proteins was performed according to Yang et al. [16].

### PCA and correlation analysis

The differentially expressed proteins derived from Mg-deficient *C. sinensis* leaves and roots were transformed for the PCA using the Unscrambler (version 10.0.1, Camo Software Inc., Woodbridge, NJ, USA). The PCA loading plots were used to determine the separation of the differentially expressed proteins based on the presence and absence of Mg in *C. sinensis* leaves and roots. In this system, we used each protein response as predictor variables, whereas treatment factor (control *versus* Mg-deficiency) was considered to be response variable. The treatment factor was introduced as separate categorical variables [reading either −1 (Mg-deficiency) or 1 (control)]. The PCA loading plots were performed in triplicate (*n* = 3).

Pearson correlation analysis was performed to determine the relationships between the differentially expressed proteins derived from Mg-deficient *C. sinensi*s leaves and roots. Pearson correlation coefficients were obtained by subjecting the data set with correlation procedure (PROC CORR) in SAS version 8.02 to determine the effect of Mg-deficiency in *C. sinensis* leaves and roots. Red and blue colors indicated a positive and negative correlation coefficient between the differentially expressed proteins derived from Mg-deficient *C. sinensis* leaves and roots. The correlation analysis was performed in triplicate (*n* = 3).

### Real time quantitative reverse transcription PCR (qRT-PCR) analysis

Total RNA was extracted from the same root and leaf samples used for 2-DE analysis using Recalcirtant Plant Total RNA Extraction Kit (Centrifugal column type, Bioteke Corporation, China). About 2.0 μg total RNA was used for first-strand cDNA synthesis using the RevertAid™ First-Strand cDNA Synthesis Kit (Thermo Scientific, Massachusetts, USA) following the manufacturer’s instructions. The resulting cDNA was diluted 50-fold using Tris-EDTA buffer (10 mM Tris, 50 mM NaCl, 1 mM EDTA, pH 7.8). Gene-specific primers were designed using Primer Software Version 5.0 (PREMIER Biosoft International, CA, USA) according to the corresponding sequences of selected proteins in *Citrus* genome (http://phytozome.jgi.doe.gov/pz/portal.html#!info?alias=Org_Cclementina). The sequences of the F and R primers used are given in Additional file 3. qRT-PCR was performed using a SYBR®Premix Ex Taq™ (Tli RNaseH Plus, TaKaRa, Japan) with the Step One Plus Real-Time System (Applid Biosystems, CA, USA) in an Eco Real-Time PCR System (Illumina, CA, USA). The cycling conditions were 15 s at 95°C, followed by 40 cycles of 95°C for 5 s, 65°C for 34 s. Samples for qRT-PCR were run in three biological replicates with two technical replicates. Relative gene expression was calculated using ddCt algorithm. For the normalization of gene expression, citrus *actin* (GU911361.1) was used as an internal standard and the leaves from control plants were used as reference sample, which was set to 1.

### Experimental design and statistical analysis

There were 40 seedlings (20 pots) per treatment in a completely randomized design. Experiments were performed with 3–10 replicates (primarily three). The same experimental samples were used for 2-DE and qRT-PCR analysis. Differences among treatments were separated by the unpaired *t*-test at *P* < 0.05 level.

### Availability of supporting data

The mass spectrometry proteomics data have been deposited to the ProteomeXchange Consortium *via* the PRIDE partner repository with the dataset identifier PXD001871.

## Additional files

Additional file 1:
**Differentially expressed proteins in Mg-deficient leaves (A) and roots (B) as compared with control ones.**
Additional file 2:
**Relative expression of three marker genes for jasmonate signalling in control and Mg-deficient roots.**
Additional file 3:
**Specific primer pairs used for qRT-PCR expression analysis.**
