# Supplementary material for: Proteomic analysis of Citrus sinensis roots and leaves in response to long-term magnesium-deficiency
Source: BMC Genomics. 2015 Mar 31;16(1):253. doi: 10.1186/s12864-015-1462-z (PMC4383213; doi:10.1186/s12864-015-1462-z)
Supplement: Additional file 1: — Differentially expressed proteins in Mg-deficient leaves (A) and roots (B) as compared with control ones. [file 12864_2015_1462_MOESM1_ESM.doc]

**Additional file 1: Differentially expressed proteins in Mg-deficient leaves (A) and roots (B) as compared with control ones.**
