# Supplementary material for: Proteomic analysis of Citrus sinensis roots and leaves in response to long-term magnesium-deficiency
Source: BMC Genomics. 2015 Mar 31;16(1):253. doi: 10.1186/s12864-015-1462-z (PMC4383213; doi:10.1186/s12864-015-1462-z)
Supplement: Additional file 2: — Relative expression of three marker genes for jasmonate signalling in control and Mg-deficient roots. [file 12864_2015_1462_MOESM2_ESM.doc]

**Additional file 2: Relative expression of three marker genes for jasmonate signalling in control and Mg-deficient roots.** Bars represent means ± SE (*n* = 4). Different letters above the bars indicate a significant difference at *P* < 0.05. All the values were expressed relative to the control leaves or roots. The sequences of the forward primers and reverse primers for actin (Ciclev10011973m|PACid:20796733), ZIM/tify-domain (Ciclev10008797m|PACid:20794681, JAZ/TIFY), MYC2 (Ciclev10011214m|PACid:20797158) and lipoxygenase 2 (Ciclev10017776m|PACid:20817453; LOX2) genes are 5'-AGAACTATGAACTGCCTGATGGC-3', 5'-CATCCAACAGAACAACTTCACCA-3', 5'-CTTCGCCGACTGATGATGCTG-3' and 5'-AGATTGTTGCATCCTCATTTCCG-3', and 5'-GCTTGGAGCAAGTGCTGTGATT-3', 5'-AGATCTCCAAAGCTACCTCCCTC-3', 5'-GAAAACCTGCCCCTGTCTTGC-3' and 5'-CCATCCTTGGCATAAGGGTAGTC-3', respectively.
