# Supplementary material for: Proteomic analysis of Citrus sinensis roots and leaves in response to long-term magnesium-deficiency
Source: BMC Genomics. 2015 Mar 31;16(1):253. doi: 10.1186/s12864-015-1462-z (PMC4383213; doi:10.1186/s12864-015-1462-z)
Supplement: Additional file 3: — Specific primer pairs used for qRT-PCR expression analysis. [file 12864_2015_1462_MOESM3_ESM.doc]

**Additional file 3: Specific primer pairs used for qRT-PCR expression analysis.**

| Spot No. | Gene | Forward primers (5´→3´) | Reverse primers (5´→3´) |
| --- | --- | --- | --- |
| L4 | *Cysteine proteinase* | TCAAATGGTCGCTATTCGTC | TCTCCTTGAACCTTATGCTCC |
| L33 | *ATP synthase CF1 alpha subunit, chloroplastic* | GCAGAAGCGTTGCGTGAAA | TATCCAGACCCCAGAAGACC |
| L44 | *Triosephosphate isomerase* | TGAGCCAGTTTGGGTCATT | TGCTGCAACTTCAGCATTG |
| L78 | *Cu Zn superoxide dismutase* | AAAATGGAAACAGGGGCTAC | ACTCCGTTGGGTCCTTGAA |
| L80 | *Phosphoglycerate kinase* | GGTTCAAAGGTCTCATCCAAG | AATCCCATCCAGCCATCA |
| L96 | *Ascorbate peroxidase* | GCTGGTTCTTGCTTCTTTCC | TTGACTCCCCACTACATTCCT |
| L102 | *S-adenosylmethionine synthetase* | TGCTCGTCTCACGGAGGTTA | TCAAGGTACTTCTCGGGGAT |
| L104 | *Aconitate hydratase 3* | ATTGGAACTGGGAAAGATGG | GGAGGCTCGTGAATGTAGGT |
| L105 | *Nucleoside diphosphate kinase, putative* | AGGGTGTAGGTGCGTTAGGT | GAAAAGATGGGACTGTGGGT |
| L110 | *ATYKT62* | AGCTGATGGAAGCGAGGAA | GCCAAATGGCAGTGTAGTGATA |
| L114 | *Glyceraldehyde-3-phosphate dehydrogenase B subunit* | TGCTGTGACTGCTCAACTTACT | GGTCTCGTTGTCCACTATTTTC |
| L117 | *Alcohol dehydrogenase* | GTGCCAAACAAAGATGATGC | TTTCTCGACCTCCAACTCCT |
| L118 | *Ribulose 1,5-bisphosphate carboxylase, partial* | CAGTTGCTGGTTCTGGCTAT | GGCATTCTGCTATTCTCCCT |
| R1 | *Proteasome subunit alpha type, putative* | GGTGCTACGCTTTGGTGAA | TCTGCCTCCTGAAGGGTTA |
| R13 | *Cysteine protease, putative* | TCTACGACGGGCTTCATTAT | TGCTGCTCCTTGACAAGTTTC |
| R85 | *Pyruvate decarboxylase, putative* | CCTTATCAACAATGGCGGGTAT | GCTCTTTGCTGGTGTCATCC |
| R95 | *Phosphoglycerate kinase* | CCTTCTGTGGCTGGTTTCCT | GAGACACCCCTTTAGATTTGG |
| R121 | *Hexokinase* | ACCGTCGTCATTGAGCATT | CCGAAAGAACTAAGAAGGGAG |
|  | Actin | AGAACTATGAACTGCCTGATGGC | GCTTGGAGCAAGTGCTGTGATT |
